# Closing the Evidence-Practice Gap: A Quality Improvement Audit of Antibiotic Prescribing Following Incision and Drainage of Superficial Skin, Pilonidal and Perianal Abscesses

**DOI:** 10.7759/cureus.115271

**Published:** 2026-08-26

**Authors:** Finian M O'Malley, Janine Bothe

**Affiliations:** 1 General Surgery, St George Hospital, Sydney, AUS

**Keywords:** abscess, antibiotic resistance, antibiotics, clinical audit, day case surgery, general surgery, incision and drainage, quality improvement projects, superficial skin abscess

## Abstract

Background

Superficial abscesses are a common presentation to emergency and surgical services, typically managed with incision and drainage (I&D). For superficial skin abscesses, evidence suggests that adjunctive antibiotic therapy reduces treatment failure and recurrence, whereas the benefit in perianal abscesses remains less certain; in either case, prescribing practices remain inconsistent. At an Australian metropolitan teaching hospital, a baseline audit revealed poor adherence to local antibiotic prescribing guidelines following I&D of superficial skin and perianal abscesses, with variability in both antibiotic choice and duration.

Method

A retrospective audit of antibiotic prescribing in 49 patients undergoing I&D of superficial skin, pilonidal or perianal abscesses over a six-week period was performed. Patients with systemic infection or sepsis precluding immediate discharge, recurrent abscesses, deep-space extension, or those requiring drain insertion were excluded. Educational interventions were then implemented, including dissemination of a concise prescribing guideline via posters, email communication, and an in-person departmental meeting. A re-audit over the following six weeks was subsequently conducted; 59 patients met the inclusion criteria.

Results

A total of 108 patients were included across both audit cycles (49 pre-intervention, 59 post-intervention). Pre-intervention, 7/49 patients (14.3%) received guideline-concordant antibiotic therapy, indicating significant under-prescribing relative to local guidelines at baseline. Following the educational intervention, this increased to 24/59 patients (40.7%), representing an absolute improvement of 26.4 percentage points (95% CI: 10.5 to 42.3; χ²=9.11, p=0.0025). Although the confidence interval is wide, reflecting the modest sample size, it excludes no effect and is consistent with a clinically meaningful improvement in prescribing behaviour. It is notable, however, that 35/59 patients (59.3%) still did not receive guideline-concordant therapy following the intervention, underscoring that substantial room for improvement remains.

Conclusion

A simple educational intervention was associated with a clinically meaningful improvement in guideline-concordant antibiotic prescribing following abscess drainage, though the 70% compliance target was not reached, highlighting the limits of single-cycle educational initiatives in achieving full guideline concordance. For superficial skin abscesses, the evidence base is robust and efforts to embed guideline awareness into clinical practice are justified. For perianal abscesses, the evidence remains conflicting, and further high-quality trial data are needed before definitive prescribing recommendations can be made.

## Introduction

Superficial abscesses are frequently encountered in both emergency and general surgical practice. For the purposes of this project, "superficial" abscesses were defined as localized, non-necrotizing skin, pilonidal or perianal abscesses without deeper anatomical extension, amenable to drainage in a single procedure. While incision and drainage (I&D) remains the primary treatment, adjunctive antibiotic therapy is recommended in many cases. Despite this, prescribing practices are often inconsistent, leading to inappropriate antibiotic selection and duration.

At a large metropolitan teaching centre, variation in antibiotic prescribing following I&D of uncomplicated abscesses was observed in routine clinical practice. This variability posed potential risks at both ends of the prescribing spectrum: under-treatment could increase treatment failure and recurrence, while inappropriate or unnecessarily prolonged courses contribute to avoidable harms, including adverse drug reactions, Clostridium difficile infection, and selection for antimicrobial resistance (AMR).

The SMART aim of this project was to increase compliance with evidence-based antibiotic prescribing guidelines to ≥70% of eligible patients receiving correct antibiotic therapy and duration within six weeks. As a quality improvement initiative, the project was structured around this practical aim; an accompanying hypothesis was specified to permit formal statistical evaluation of the intervention. We hypothesised that a simple, low-cost educational intervention disseminating the local prescribing guideline would significantly increase the proportion of patients receiving guideline-concordant antibiotic therapy following I&D, relative to baseline.

A six-week window was chosen for two reasons: it aligns with the rotation cycle of junior medical staff, who are responsible for the majority of discharge prescribing, ensuring that both audit cycles captured a comparable cohort of prescribers and that the post-intervention audit was completed before the effect was diluted by staff turnover; and it was the minimum period expected to accrue a sufficient number of eligible cases to detect a meaningful change in prescribing concordance.

There is a growing body of evidence supporting the use of adjunctive antibiotic therapy following I&D of superficial skin abscesses. A systematic review and meta-analysis of randomised controlled trials demonstrated that antibiotics significantly reduce treatment failure rates (8% with antibiotics vs 16% with placebo; OR 2.3, 95% CI 1.8-3.1) [1]. These findings are supported by a larger network meta-analysis involving over 4,000 patients, which also demonstrated reductions in recurrence rates with antibiotic therapy [2].

Randomised controlled trial data further suggest that the benefit of antibiotics is consistent across a range of clinical presentations, including variation in lesion size, causative organism, and patient comorbidities [3-5]. In addition to reducing treatment failure, antibiotics have been shown to reduce recurrence, with one meta-analysis reporting recurrence rates of 7.6% versus 14.5% within one month [2]. Collectively, these findings provide a strong evidence base supporting adjunctive antibiotic use in superficial skin abscesses.

In contrast, the role of antibiotics in the management of perianal and anorectal abscesses remains less clearly defined. A systematic review and meta-analysis reported a reduction in fistula formation in patients receiving antibiotics following drainage (16% vs 24%; OR 0.64, 95% CI 0.43-0.96) [6]. However, this analysis included a limited number of studies with significant heterogeneity and risk of bias. Individual randomised controlled trials have produced conflicting results, with some demonstrating no benefit in reducing fistula formation [7], while others have shown a moderate benefit [8]. Consequently, the overall evidence base remains inconclusive.

This is reflected in differences between clinical guidelines. Local institutional guidelines recommend routine administration of a short course of oral antibiotics following I&D of superficial skin, pilonidal and perianal abscesses. In contrast, the American Society of Colon and Rectal Surgeons recommends a more selective approach, reserving antibiotic therapy for patients with systemic infection, significant cellulitis, or specific comorbidities [9].

Despite an expanding evidence base, translation into clinical practice remains inconsistent. Variability in prescribing behaviour likely reflects both gaps in clinician awareness and the need for more conclusive evidence of benefit, particularly in perianal disease. Quality improvement approaches are therefore well suited to address this evidence-practice gap and promote more consistent, guideline-adherent care.

## Materials and methods

Patients were identified retrospectively from theatre records over a six-week baseline period (7 July to 17 August 2025, 42 days), using a report generated from the theatre electronic medical record system. For each patient, primary outcome data were extracted from the theatre electronic medical record by a single investigator using identical criteria. The investigator was not blinded to pre- or post-intervention status, as the audit periods were sequential and identifiable from theatre record dates. The primary outcome was binary, assessed against fixed criteria for antibiotic agent and duration; no independent second extraction was performed.

Patient demographic data were not collected. As an initial audit cycle intended to establish baseline practice and test the feasibility of a low-cost educational intervention, data collection was deliberately restricted to the minimum dataset required to determine the primary outcome, minimising the data-handling burden and enabling rapid completion of both cycles within the constraints of routine clinical service. These are acknowledged as limitations in the discussion.

Abscess site was additionally not recorded because the audit standard was uniform: local guidelines recommend the same approach, a five-day course of oral antibiotics, following drainage of superficial skin, pilonidal and perianal abscesses, so guideline concordance was defined identically across all abscess types. The appropriateness of a uniform standard, given the contested evidence base in perianal disease, is considered in the discussion.

The educational intervention was delivered on 18 August 2025, immediately following the baseline period. The audit was then repeated using the same data collection methodology over a second six-week period (18 August to 28 September 2025, 42 days). Categorical variables were analysed using the chi-squared (χ²) test, performed using GraphPad QuickCalcs (GraphPad Software, San Diego, CA, USA). Statistical significance was set at p<0.05. Absolute differences in proportions are reported with 95% confidence intervals (CIs).

Inclusion criteria

All patients at the hospital undergoing I&D of superficial skin abscess, pilonidal abscess or perianal abscess in the operating theatres during the six-week audit period were included.

Exclusion criteria

Patients were excluded if they required surgical drain insertion, had undergone previous I&D at the same site (recurrent abscess), had evidence of deeper anatomical extension (for example, into the ischioanal or supralevator space), or had clinical signs of systemic infection or sepsis such that they were not safe for discharge immediately following drainage. The audited cohort therefore comprised patients discharged directly after drainage, in whom antibiotic prescribing at discharge was applicable.

Primary outcome measure

The primary outcome measure is the proportion of patients receiving guideline-concordant antibiotic therapy according to local antibiotic guidelines (Table 1). Prescriptions were classified as guideline-concordant only if both the antibiotic agent and the five-day duration matched the local guideline (Table 1); any deviation in agent or duration, including omission of antibiotics, was classified as non-concordant. Antibiotic dose was not assessed.

**Table 1:** Local antibiotic guidelines for superficial skin, pilonidal or perianal abscesses (five-day duration of prescription)

Condition	First-Line Antibiotics	Penicillin Allergy Alternatives
Superficial Skin Abscess	Oral Flucloxacillin 500 mg QID or Cephalexin 250 mg QID	Oral Co-trimoxazole (Bactrim) 1 BD or Clindamycin 150 mg QID
Pilonidal/Perianal Abscess	Oral Cephalexin 500 mg QID	Oral Co-trimoxazole (Bactrim) 1 BD or Clindamycin 150 mg QID

Intervention

The educational intervention was designed as a simple, low-cost educational strategy to improve clinician awareness of local antibiotic prescribing guidelines and supporting evidence.

**Figure 1:**
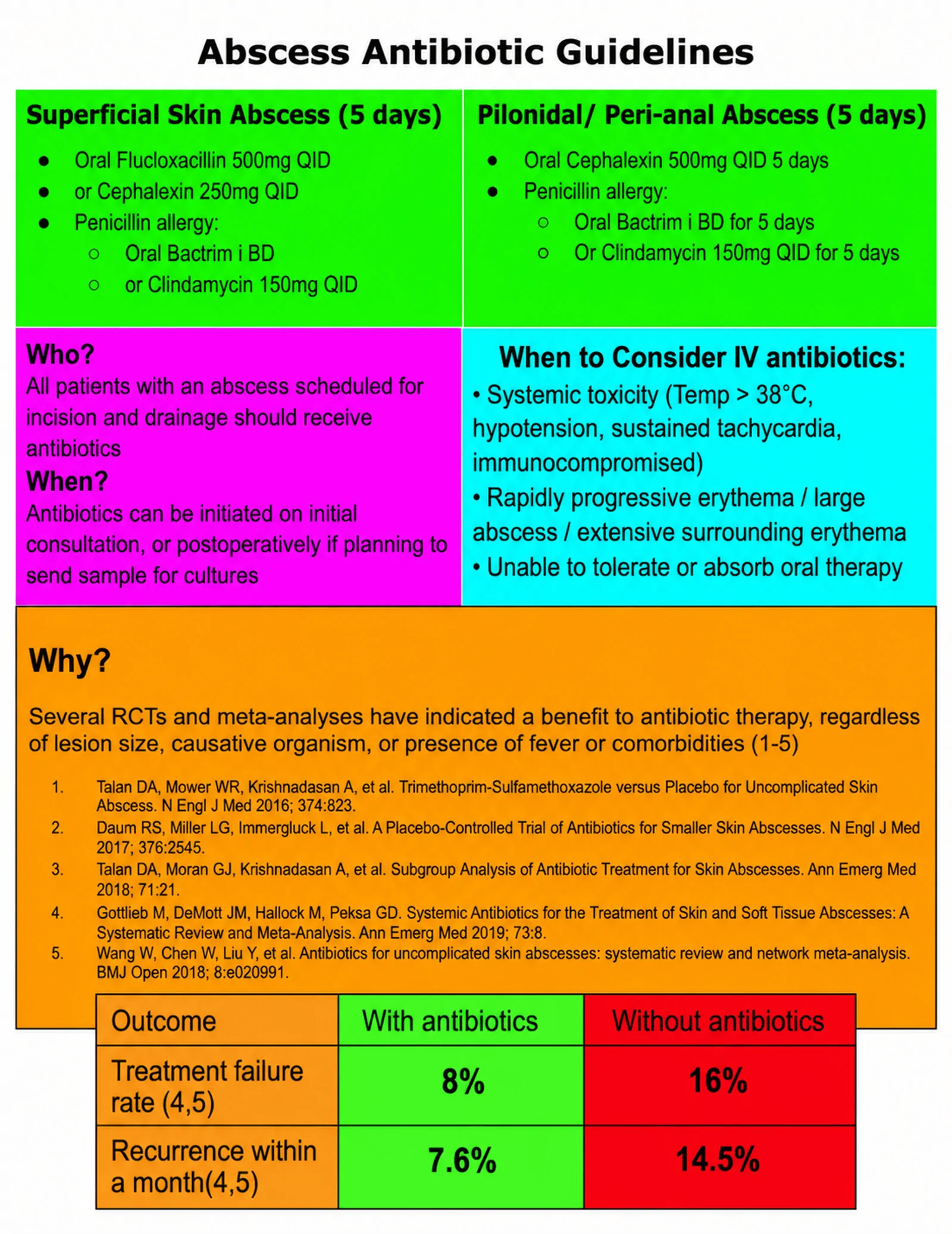
Interventional poster: local antibiotic guidelines for superficial skin, pilonidal or perianal abscesses (five-day duration of prescription) Image Credit: Dr Finian O'Malley The figure was prepared using Microsoft Word Interventional poster: local antibiotic guidelines for superficial skin, pilonidal or perianal abscesses (five days duration of prescription). References cited within the poster correspond to ref [1-5] (poster reference 1 = ref [3]; 2 = ref [5]; 3 = ref [4]; 4 = ref [1]; 5 = ref [2])

A concise educational poster (Figure 1) was developed and displayed in the emergency department treatment areas and the offices of the surgical teams at the start of the second data collection period on August 18th. It was distributed digitally by email and a secure messaging application to all surgical registrars and presented at the general surgery grand rounds meeting the following day on August 19th, attendance at which is mandatory for surgical staff. However, attendance varies with clinical availability, so the exact number of clinicians exposed to each component of the intervention is not known. The target audience comprised all surgical medical staff and emergency department clinicians prescribing at discharge following I&D.

The poster presented information on antibiotic type and duration guidelines and the supporting evidence base. High-visibility formatting was used to maximise uptake in busy clinical environments. It was anticipated that improving visibility and accessibility of guidelines would positively influence prescribing behaviour. Sustainability was considered at the design stage, with plans to embed the intervention through continued display of materials and integration into staff induction processes. 

## Results

A total of 108 patients were included across both audit cycles (49 pre-intervention, 59 post-intervention).

Primary outcome

**Figure 2:**
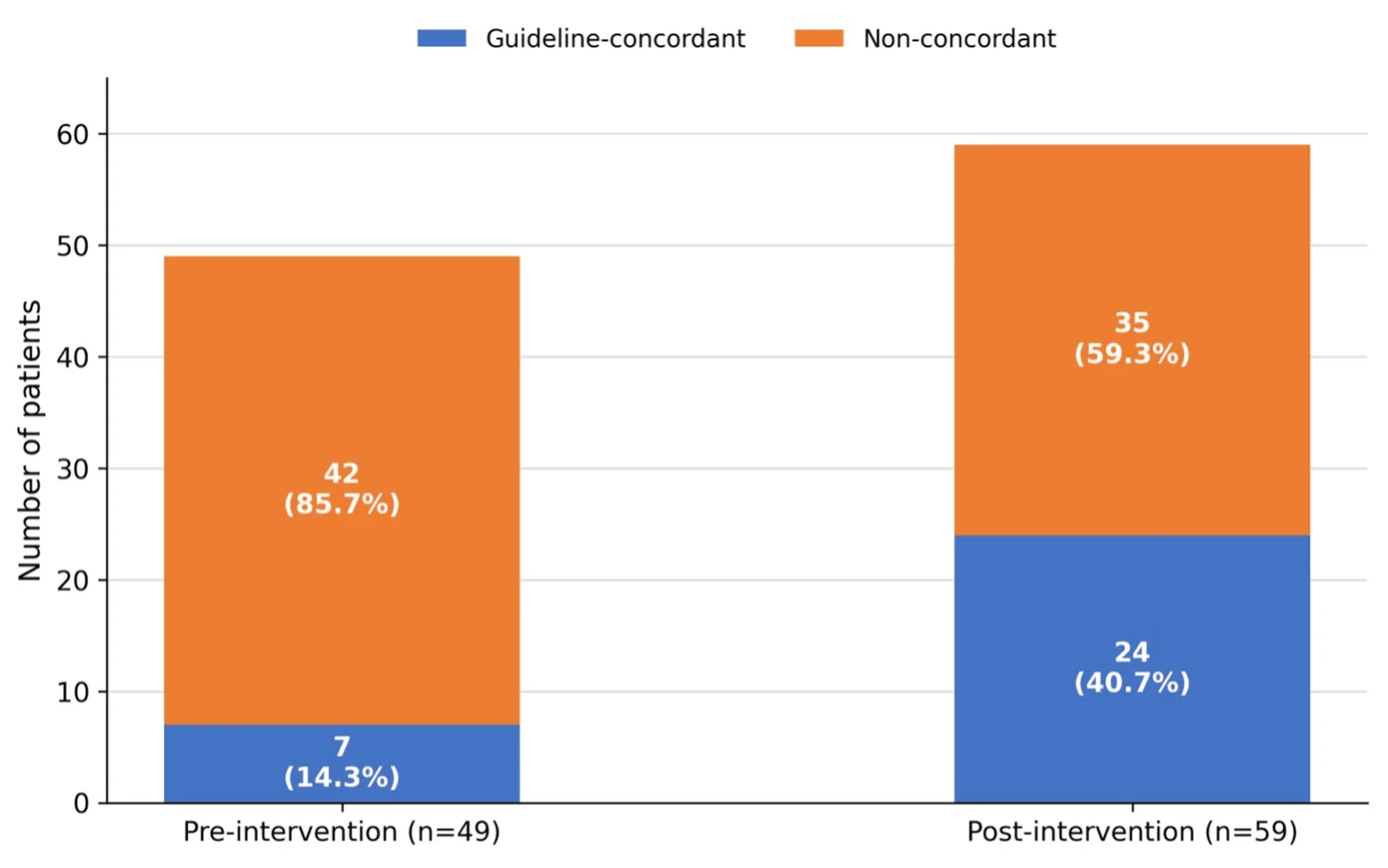
Number of patients receiving guideline-concordant antibiotic therapy pre- and post-educational intervention Stacked bars show the number of patients receiving guideline-concordant (blue) and non-concordant (orange) therapy, with counts and percentages labelled. Image Credit: Dr Finian O'Malley The figure was prepared using Microsoft Excel

Pre-intervention, 7/49 patients (14.3%) received guideline-concordant antibiotic therapy. Post-intervention, this rose to 24/59 (40.7%), an absolute increase of 26.4 percentage points (95% CI: 10.5 to 42.3; χ²=9.11, p=0.0025) (Table 2; Figure 2).

**Table 2:** Proportion of patients receiving guideline-concordant antibiotic therapy The p-value is considered significant when p<0.05 (denoted with '*' in the table).

Outcome	Pre-intervention	Post-intervention	Absolute difference (95% CI)	χ²	p-value
Guideline-concordant antibiotic therapy	7/49 (14.3%)	24/59 (40.7%)	26.4% (10.5 to 42.3)	9.11	0.0025*

Although the CI is wide, reflecting the modest sample size, the lower bound of 10.5 percentage points nonetheless represents a clinically meaningful improvement in prescribing behaviour. These findings suggest that the educational intervention was associated with a significant positive change in antibiotic prescribing practice.

It is notable, however, that even following the intervention, 35/59 patients (59.3%) still did not receive guideline-concordant therapy, indicating that while meaningful progress was achieved, substantial room for improvement remains. These findings are explored further in the context of existing literature in the discussion.

## Discussion

In this project, a simple, low-cost educational intervention was associated with improved adherence to evidence-based antibiotic prescribing guidelines following I&D of superficial abscesses. Guideline concordance rose by 26.4 percentage points following the intervention, a statistically significant and clinically meaningful improvement. However, the SMART aim of 70% compliance was not achieved, and the majority of patients in the post-intervention period still did not receive guideline-concordant therapy, indicating that meaningful room for improvement remains.

Key lessons include that clinician awareness appears to be an important influence on prescribing behaviour, that accessible and concise guidance facilitates adherence, and that low-resource interventions, requiring no dedicated funding and readily replicable in other centres, can achieve meaningful improvements.

Several limitations should be acknowledged. This was a single-centre study with a relatively small sample size, limiting generalisability. As an uncontrolled pre-post design, this project cannot establish causality; secular trends or co-occurring changes in practice may have contributed to the observed improvement. The short follow-up period precluded assessment of long-term sustainability, and potential confounding factors, including staff turnover, may have influenced results.

Data were extracted by a single investigator who was not blinded to pre- or post-intervention status; although the primary outcome was binary and assessed against fixed, objective criteria, no independent second extraction was performed to verify accuracy. Prescriptions were variably issued electronically or on paper, and paper prescriptions were not accessible through the electronic medical record, which may have introduced incomplete ascertainment of the primary outcome, although this would be consistent across both cycles.

Demographic and case-level data were not collected, and consequently, the pre- and post-intervention cohorts could not be formally compared. In particular, the abscess site was not recorded, so concordance could not be stratified by the abscess type, and a shift in case mix between cycles cannot be excluded as a contributor to the observed change. This is a notable limitation given that the evidence base, and therefore the interpretation of non-concordance, differs between superficial skin and perianal abscesses: non-concordance in perianal cases may represent deliberate clinical judgement rather than unfamiliarity with the guideline, and these cannot be distinguished in our data. However, the hospital guidelines are clear in their recommendation of a five-day course of oral antibiotics following drainage of all three abscess types, so guideline concordance was defined identically across the audited cohort; whether a uniform standard is appropriate given the contested perianal evidence is considered below.

Future audit cycles should record abscess site and report concordance separately for superficial skin, pilonidal and perianal abscesses, alongside prescriber rationale for deviations; these data would also inform any revision of the local guideline. The number of clinicians exposed to each component of the intervention was not recorded, limiting reproducibility and precluding assessment of intervention reach. Guideline concordance is a process measure and a surrogate for prescribing quality; a prescription may be guideline-concordant yet not optimal for an individual patient, and clinical appropriateness in individual cases was not assessed. Finally, clinician awareness that prescribing practice was subject to audit may itself have influenced behaviour, independent of the educational content (Hawthorne effect).

Future studies should evaluate the impact of improved prescribing on patient outcomes. This is important because uncertainty remains regarding the optimal role of antibiotics, particularly in perianal abscesses: existing evidence suggests potential benefit in reducing fistula formation but is limited by heterogeneity and conflicting trial results [6-8], and ongoing clinical trials are expected to provide further clarity [10,11].

Although not directly examined in this study, these findings should also be interpreted within the wider context of antimicrobial stewardship. Stewardship describes coordinated efforts to optimise antimicrobial use, promoting the right agent, dose, route, and duration in order to improve patient outcomes while minimising harms such as toxicity, Clostridium difficile infection, and the emergence of AMR [12]. Crucially, stewardship is concerned with appropriate prescribing rather than simply maximising or minimising antibiotic use, and these findings are consistent with the broader stewardship literature demonstrating that educational strategies can improve guideline compliance in hospital settings [13,14]. A systematic review and meta-analysis of stewardship objectives found that guideline-adherent empirical therapy was associated with a 35% relative risk reduction in mortality, underscoring that appropriate prescribing has direct patient benefit beyond resistance mitigation alone [15].

In this context, an intervention designed to increase prescribing compliance is caught in a genuine clinical conundrum: encouraging greater antibiotic use on the basis of weak or conflicting evidence risks driving avoidable antimicrobial exposure, which carries its own patient and population-level harms. These harms are not trivial. A systematic analysis estimated that approximately 1.27 million deaths were directly attributable to bacterial AMR in 2019, with a further 4.95 million deaths associated with resistant infections, a burden exceeding that of HIV/AIDS or malaria [16]. Updated modelling projects that without effective intervention, an estimated 39.1 million deaths will be directly attributable to AMR cumulatively between 2025 and 2050 [17]. Against this backdrop, promoting routine antibiotic use where the evidence of benefit is uncertain represents a real ethical tension that quality improvement initiatives in this space must acknowledge as a cautionary consideration for future practice.

This reinforces the importance of condition-specific guidelines grounded in high-quality evidence, so that improved compliance does not inadvertently promote unnecessary prescribing. Evidence from ongoing trials will be essential in defining the appropriate balance between benefit and harm when considering adjunctive antibiotic therapy following perianal abscess drainage [10,11].

## Conclusions

In this quality improvement project, a simple, low-cost educational intervention was associated with a substantial improvement in guideline-concordant antibiotic prescribing following abscess drainage, consistent with clinician awareness being an important influence on prescribing behaviour. However, compliance remained below the pre-specified target, underscoring that passive educational initiatives alone are insufficient to achieve full guideline concordance. Posters, email and departmental teaching rely on clinicians recalling and applying guidance at the point of prescribing, and are readily diluted by workload and staff turnover. Sustained improvement is likely to require structural measures that make the guideline-concordant choice the default: for example, embedding prescribing prompts or pre-configured order sets within the electronic medical record, integrating the guideline into surgical induction, and repeated audit cycles to monitor performance.

For superficial skin abscesses, the evidence supporting adjunctive antibiotic therapy is robust, and efforts to increase guideline awareness are both justified and necessary. For perianal abscesses, the evidence base remains limited and conflicting, and further high-quality trial data are needed before prescribing recommendations can be made with confidence. Aligning quality improvement initiatives with emerging evidence in this area will be essential to ensure that improved compliance translates into genuine patient benefit rather than inadvertent promotion of unnecessary antibiotic use.
